# Prognostic impact of tumor infiltrating lymphocytes on patients with metastatic urothelial carcinoma receiving platinum based chemotherapy

**DOI:** 10.1038/s41598-018-25944-1

**Published:** 2018-05-10

**Authors:** Hui-Shan Huang, Harvey Yu-Li Su, Pei-Hsu Li, Po-Hui Chiang, Cheng-Hua Huang, Chien-Hsu Chen, Meng-Che Hsieh

**Affiliations:** 1 Department of Pathology, Kaohsiung Chang Gung Memorial Hospital and Chang Gung University College of Medicine, Kaohsiung, Taiwan; 2 Division of Hematology-Oncology, Department of Internal Medicine, Kaohsiung Chang Gung Memorial Hospital and Chang Gung University College of Medicine, Kaohsiung, Taiwan; 3 Division of Urology, Department of Surgery, Kaohsiung Chang Gung Memorial Hospital and Chang Gung University College of Medicine, Kaohsiung, Taiwan

## Abstract

The impact of tumor infiltrating lymphocytes (TILs) on survival was confirmed in various cancer types. Our study aims to investigate the prognostic role of TILs on survival in patients with metastatic urothelial carcinoma (mUC) receiving platinum based chemotherapy. Patients who were diagnosed to have pathologically proved mUC between 1997 and 2016 and received palliative chemotherapy with platinum based regimen were recruited into our study. Kaplan-Meier curves and Cox regression analysis were constructed for overall survival (OS). A total of 259 mUC patients were enrolled into our study with median age 63 years and median follow-up visit 13.5 months. Of these patients, 179 (69%) had intense TILs and 80 (31%) had non-intense TILs. The median OS were 15.7 vs. 6.7 months (*P* = < 0.001) for patients with intense TILs and non-intense TILs, respectively. Subgroup analysis showed that TILs was both prognostically significant no matter for urothelial carcinoma of bladder and upper tract urothelial carcinoma. Multivariate analysis showed that TILs were strongly prognostic factors related to OS. Our study suggested mUC patients with intense TILs were independently associated with survival. Based on our study, TILs is clinically useful for outcomes anticipation and risk stratification, as well as patients counseling.

## Introduction

Urothelial carcinoma is the leading malignancy of genitourinary tract^1^. The only curative treatment is radical surgery. However, disease recurrence is usually developed in the future, resulting in poor prognosis. Palliative chemotherapy with platinum based regimen remains the golden standard for patients with advanced, recurrent metastatic urothelial carcinoma (mUC)^2^. For fit patients with mUC, Methotrexate/vinblastine/doxorubicin/cisplatin(MVAC) and gemcitabine/cisplatin are two commonly used regimens^3^. The outstanding phase III study conducted by von der Maase *et al*. demonstrated that the median survival was 14.0 months for GC and 15.2 months for MVAC (hazard ratio [HR]: 1.09; 95% CI: 0.88 to 1.34; P = 0.66), respectively and concludes that GC provided a similar survival advantage as that of MVAC with a better safety profile as well as a improved tolerability^4^. For unfit patients, carboplatin was used instead of cisplatin in case of renal toxicity. EORTC study 30986 exhibited the efficacy of carboplatin based regimen with a median survival ranging from 8.1 to 9.3 months^5^. Although recent advances of anti-cancer modality, the mortality rate has not changed over the past two decades substantially with the median overall survival of no more than 15 months^6^. Several publications made great efforts to explore the reliable prognostic factors for outcomes prediction. It has been well understanding that histological variants^7^, lymphovascular invasion^8^, positive surgical margin^9^, hepatic metastases^10–12^ and more than one metastatic site^12^ were all significant to OS. However, these prognosticators merely based on tumor behaviors, excluding the immunologic reaction between host and tumor.

Undoubtedly, tumor microenvironment is closely related to treatment outcomes^13^. There is increasing evidence that the interaction between immune cells and tumor cells is critical for the development and progression of cancer^14^. It is worth noting that tumor-infiltrating lymphocytes (TILs) play an important role in that situation. TILs are frequently found in tumors, suggesting that tumors trigger an immune response in the host^15^. The impact of TILs on survival was confirmed in various cancer types^16–21^. Furthermore, the association between TILs and treatment response was also emphasized in several publications^22,23^. Given that the understandings of the relationship between TILs and prognosis in patients with mUC were limited, there is an urgent need to determine the clinical relevance of TILs in patients with mUC. Therefore, our study aims to identify the prognostic impact of TILs on survival in patients with mUC receiving platinum based chemotherapy.

## Result

### Baseline Characteristics

The median age was 63 years and median follow-up visit was 13.5 months. As for location of primary tumor, 166 patients had upper tract urothelial carcinoma (UTUC) and 93 patients have urothelial carcinoma of bladder (UCB). Table 1 summarized the clinical characteristics of our patients. The median age at diagnosis was 62 years. The majority of our patients were male in gender (65%), with a good ECOG performance status (0–1) (81%) and fit renal function (68%). Nearly two third of our patients had primary tumor in their upper urinary tract. Most patients (91%) underwent nephroureterectomy or cystectomy for tissue proof while the remains (9%) received transurethral resection of bladder tumor. After metastasis, half of our patients had single metastatic site and 81% received cisplatin based chemotherapy as first line treatment. All these specimens were sent for TILs assessment. After stratified by TILs, 179 patients (69%) had intense TILs and 80 (31%) had non-intense TILs. As compared to those with non-intense TILs, patients with intense TILs tended to have more female in gender (42% vs. 20%, *P* = 0.052), older ages (65% vs. 45%, *P* = 0.073) and single metastatic site (56% vs. 35%, *P* = 0.068). Apart from these, there were no significant differences in performance status, renal function, location of primary tumor, first-line chemotherapy regimen for metastatic disease and cycles of first-line chemotherapy.

**Table 1:** Clinical characteristics of 259 patients with metastatic urothelial carcinoma.

	Intense TIL		Non-intense TIL		*p* value
	*N* = 179 (%)		*N* = 80 (%)		
Gender					0.052
Male	103	58%	64	80%	
Female	79	42%	16	20%	
Age					0.073
≤60	62	35%	44	55%	
>60	117	65%	36	45%	
ECOG Performance status					0.548
0–1	142	79%	68	85%	
≥2	37	21%	12	15%	
Clearance of creatinine (mL/min)					0.378
≥60	116	65%	60	75%	
<60	63	35%	20	25%	
Location of primary tumors					0.238
Upper urinary tract	122	68%	44	55%	
Bladder	57	32%	36	45%	
Previous radical surgery					0.657
Yes	167	93%	69	86%	
No	12	7%	11	14%	
Number of metastatic sites					0.068
1	101	56%	28	35%	
>1	78	44%	52	65%	
First-line chemotherapy regimen					0.865
Cisplatin based	146	82%	65	81%	
Carboplatin based	33	18%	15	19%	
Cycles of first-line chemotherapy					0.178
<3	37	21%	28	35%	
≧3	142	79%	52	65%	

TIL, tumor infiltrating lymphocyte; UC, urothelial carcinoma; NLR, neutrophil to lymphocyte ratio.

### Survival outcomes

**Figure 1:**
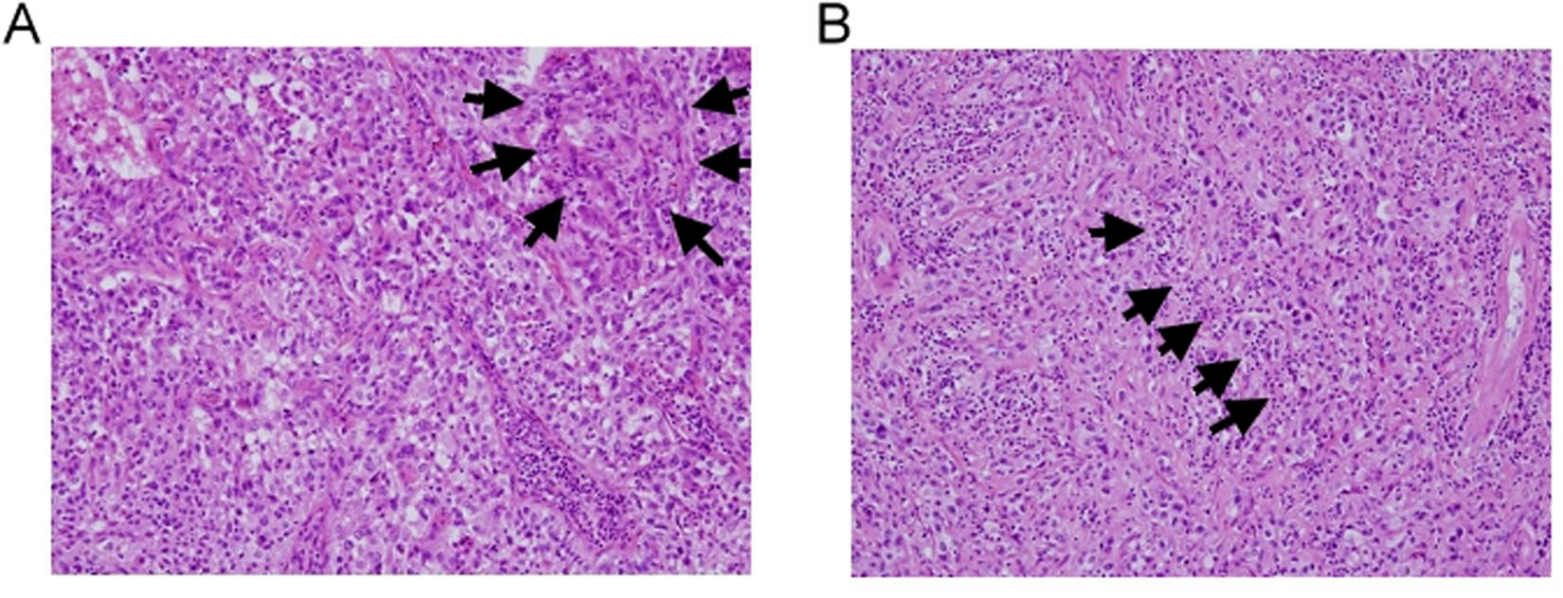
Kaplan-Meier overall survival curve of 259 patients with mUC, stratified by TILs.

**Figure 2:**
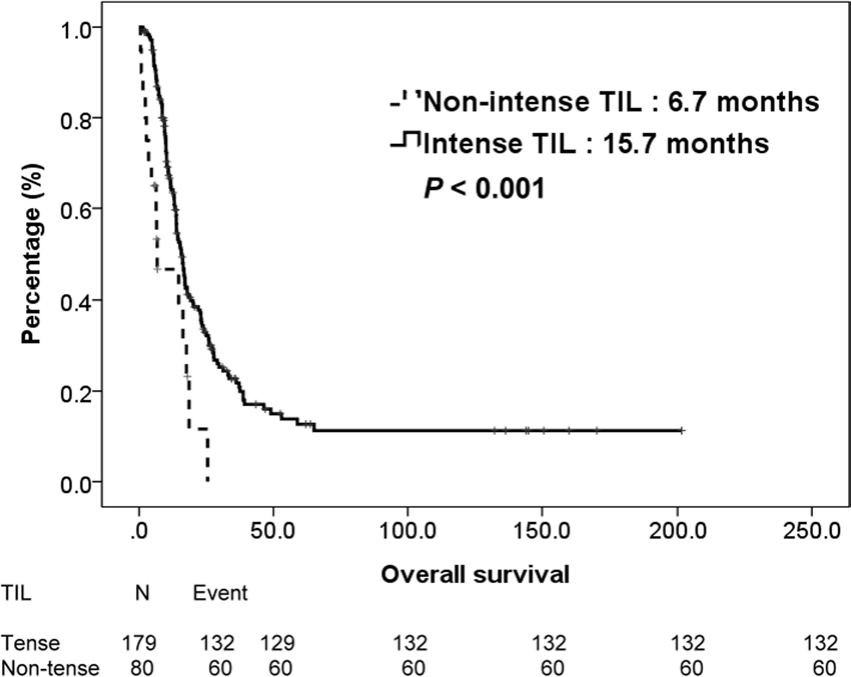
Kaplan-Meier overall survival curve of patients with UCB and UTUC, stratified by TILs.

During this period, 192 (74%) patients died and tumor was the major reason of their death. All patients were initially divided into two groups, stratified by TILs. The median OS were 15.7 vs. 6.7 months (*P* = < 0.001) for patients with intense TILs and non-intense TILs, respectively. The survival curves of intense TILs and non-intense TILs were depicted in Fig. 1. Then, we categorized all patients into UTUC group and UCB group according to their primary tumor location. For patients with UCB, the median OS were 14.0 vs. 6.5 months (*P* = 0.007) for patients with intense TILs and non-intense TILs, respectively. For patients with UTUC, the median OS were 16.4 vs. 14.7 months (*P* = 0.023) for patients with intense TILs and non-intense TILs, respectively. Figure 2 plotted the survival curves of UCB and UTUC, stratified by TILs.

### Univariate and multivariate Cox regression analyses

The univariate and multivariate analyses of OS in all patients are shown in Tables 2 and 3. In univariate analysis, age (≦60 vs. >60), gender (female vs. male), performance status (≦1 vs. >1), location of primary tumor (UTUC vs. UCB), number of metastatic sites (1 vs. ≧2) and TILs (intense vs. non-intense) had statistically impact. Moreover, TILs was classified into sTILs and itTILs according to the predominant area of lymphocyte infiltration. Subgroup analysis showed sTILs has a strong significance for survival (*P* = < 0.001), rather than itTILs (*P* = 0.148). After adjustment of confounding factors, multivariate analysis showed that female (HR: 0.65, 95% CI: 0.45–0.93, *P* = 0.020), performance status ≦1 (HR: 0.52, 95% CI: 0.34–0.79, *P* = 0.003), single metastatic sites (HR: 0.64, 95% CI: 0.46–0.89, *P* = 0.009) and TILs (HR: 0.41, 95% CI: 0.23–0.74, *P* = 0.003) were strongly prognostic factors related to OS. Age and location of primary tumor failed to be significant with OS in the final fitted Cox model.

**Table 2:** Univariate Cox regression analysis of parameters associated with overall survival.

Variables	Univariate	
	HR (95% CI)	*P* value
Age, ≦60 vs. >60	0.71 (0.51–1.00)	0.050
Gender, Female vs. Male	0.64 (0.45–0.89)	0.009
ECOG PS, ≦1 vs. >1	0.61 (0.42–0.89)	0.010
Renal function, ≧60 vs. <60	0.92 (0.65–1.30)	0.626
UTUC vs. UCB	0.70 (0.50–0.98)	0.036
Number of metastatic site, 1 vs. >1	0.65 (0.47–0.90)	0.009
Chemotherapy, cisplatin vs. carboplatin	0.61 (0.27–1.39)	0.237
TIL, intense vs non-intense	0.41 (0.24–0.71)	<0.001
Stroma TIL, intense vs non-intense	0.30 (0.19–0.48)	<0.001
Intratumor TIL, intense vs non-intense	0.79 (0.57–1.09)	0.148

ECOG PS = Eastern Cooperative Oncology Group performance status, UTUC = upper tract urothelial carcinoma, UCB = urothelial carcinoma of bladder, PUC = pure urothelial carcinoma, VUC = variant urothelial carcinoma, TIL = tumor-infiltrating lymphocyte, HR = hazzard ratio, CI = confidence interval.

**Table 3:** Multivariate Cox regression analysis of parameters associated with overall survival.

Variables	Univariate	
	HR (95% CI)	*P* value
Age, ≦ 60 vs. >60	0.72 (0.49–1.06)	0.094
Gender, Female vs. Male	0.65 (0.45–0.93)	0.020
ECOG PS, ≦ 1 vs. >1	0.52 (0.34–0.79)	0.003
UTUC vs. UCB	0.71 (0.50–1.00)	0.050
Number of metastatic site, 1 vs. >1	0.64 (0.46–0.89)	0.009
TIL, intense vs non-intense	0.41 (0.23–0.74)	0.003

ECOG PS = Eastern Cooperative Oncology Group performance status, UTUC = upper tract urothelial carcinoma, UCB = urothelial carcinoma of bladder, PUC = pure urothelial carcinoma, VUC = variant urothelial carcinoma, TIL = tumor-infiltrating lymphocyte, HR = hazzard ratio, CI = confidence interval.

## Discussion

To the best of our knowledge, this is the first study focusing on the prognostic impact of TILs on survival in patients with mUC receiving platinum based chemotherapy. Most recent publications demonstrated that TILs predicts a favorable prognosis in patients with organ confined urothelial carcinoma^24,25^. Our study suggested mUC patients with intense TILs were independently associated with better outcomes. Subgroup analysis showed that TILs was both significant for mUC patients no matter for primary tumor origin from UTUC or UCB. Furthermore, after adjustment of confounding factors, TILs remained a significant prognostic factor for OS in mUC patients. When categorizing TILs according to the area of lymphocyte infiltration, we disclosed that sTILs is more crucial than itTILs for survival of patients with mUC. This conclusion is consistent with previous literatures with regard to other types of cancers^26,27^. Thus, based on our conclusion, TILs might be clinically useful for outcomes anticipation and risk stratification, as well as patients counseling.

Several studies had demonstrated that TILs has prognostic significance in patients with various types of cancers, including breast, melanoma, lung, ovarian and anal caner *et al*.^16–21^, as well as mUC in our study. Furthermore, TILs has also shown to be associated with response to chemotherapy and prognosis^28^. The explanation of this observation remains undetermined. Current evidences showed that TILs represents an interaction between our immune system and tumor microenvironment. All immune cells may be present in the tumor microenvironment, including macrophages, neutrophil granulocytes, dendritic cells, mast cells, natural killer cells, naive and memory B lymphocytes and effector T cells (T helper cells; regulatory T cells; and cytotoxic T cells)^29^. The area infiltrated is quite widespread and can be located in the central zone of tumor, invasive margin, or peripheral tumor stroma. The immune contexture is composed of the immune cell type, density, and location, as well as the function and shaped with chemokines and cytokines^30^. As the advances of immuno-oncolgy, it is necessary for better understanding of the immunologic characteristics of the microenvironment and development of therapeutic strategies that might be beneficial to these patients^31^. Our study provided a rational toward the investigation of relationship between tumor microenvironment and prognosis in patients with mUC.

Several molecular on the surface of TILs have also been investigated. Liu *et al*. assessed FOXP3+ TILs by immunohistochemistry on tissue microarrays constructed from a well-defined cohort of 3992 breast cancer patients and found that the prognostic value of FOXP3+ TILs in breast cancer differs depending on their ER and HER2 expression status and CD8+ T-cell infiltration^32^. Webb *et al*. investigated TILs in a large collection of primary ovarian tumors and confirmed that CD103+ TILs is strongly associated with patient survival in ovary cancer, which may serve as a useful marker for enriching the most beneficial subsets for immunotherapy^19^. Djenidi *et al*. analyzed TILs on 126 lung cancer patients by multicolor flow cytometry and demonstrated that CD8+CD103+ TILs is a prognostic factor for survival^33^. Zhang *et al*. evaluated 162 specimens of upper tract urothelial carcinoma and reported PD-L1+ TILs independently predicted longer survival^34^. Wang *et al*. also estimated CD8+CD103+ TILs in patients with bladder cancer and suggested that CD8+CD103+ TILs might have a significant role in tumor immunity^25^. Patschan *et al*. reviewed 296 tissue microarray of muscle invasive bladder cancer and reported that a higher CD68/CD3 ratio of TILs was correlated with a bad prognosis^35^. Furthermore, Bellmunt *et al*. reviewed 160 specimens of mUC and concluded that PD-L1+TILs was significantly associated with longer survival^36^. In this study, we presented the prognostic role of TILs on patient of mUC. Further evaluation of molecular on TILs is ongoing.

With recent approvals for immune checkpoint inhibitors (ICI) such as cytotoxic T lymphocyte associated antigen 4 inhibitors and programmed cell death protein 1 inhibitors in caners harboring programmed death ligand 1 (PD-L1) overexpression, microsatellite instability and high mutation load, many questions regarding the biomarker for the optimal use of ICI that block these pathways are raising^23^. At the beginning, the expression of PD-L1 on immune cells or tumor cells was thought to be most reliable. However, evidences were not solid yet. TILs were an emerging prognosticator with encouraging results. Tomioka *et al*. investigated the status of TILs and PD-L1 in patients with triple negative breast cancer and found that patients with low TILs had higher probability of PD-L1 expression which could be the therapeutic target for ICI^22^. Loi *et al*. also focused on TILs in the residual disease of triple-negative breast cancers after neoadjuvant chemotherapy and demonstrated that MEK inhibition upregulated PD-L1 expression in TNBC cells with lower TILs, suggesting the possible combination use of MEK inhibitor and ICI^37^. Moreover, new or increased TILs following anti-PD1 therapy in melanoma with initial low TILs have also been observed which resulted in tumor regression^38,39^. Base on abovementioned evidences, the importance of TILs should be emphasized and hopefully can be a strong predictor of ICI.

There are several potential limitations in our work, which are inherent to any retrospective studies. In this present study, we only recruited mUC patients after prior radical surgery. Therefore, our result can not be applied to patients with de novo mUC. Furthermore, a single institutional experience, divergent treatments for metastatic disease and irregular follow-up interval also limit our study. In spite of a retrospective study with inevitable selection bias, we emphasize the significant impact of TILs in patient with mUC after prior radical surgery. Further prospective, multi-institutional studies are warranted to confirm our observations.

## Conclusions

In conclusion, TILs serves as a significant prognostic factor in patients with recurrent mUC after prior radical surgery. Our study suggested mUC patients with intense TILs were independently associated with lower NLR and better OS. Subgroup analysis showed that TILs was both significant for mUC patients with primary tumor origin from UTUC and UCB. Our findings highlight the clinical value of TILs which may useful for outcomes anticipation and risk stratification, as well as patients counseling.

## Materials and Methods

### Patients

This retrospective study was approved by the Institutional Review Board of Kaohsiung Chang Gung Memorial Hospital and informed consent was exempt from requiring. All methods were performed in accordance with the relevant guidelines and regulations. No datasets were generated or analyzed during the current study. This study was performed by histopathological analyses of tissue samples obtained from patients at our institution. Patients who were diagnosed to have pathologically proved mUC between 1997 and 2016 were recruited into our study. Clinical characteristic of our patients were collected by chart review. All patients have pathologically confirmed urothelial carcinoma. Perioperative chemotherapy and radiotherapy were permitted in our study. Exclusion criteria were not pathologically confirmed, insufficient specimens for TILs assessment and non-platinum based chemotherapy for metastatic disease. The consort flow diagram of our study was presented as Supplementary Figure 1. Initially, 308 mUC patients were identified. Of them, 22 specimens generating from biopsy were too small to perform TILs assessment and 11 surgical specimens can not be obtained. Meanwhile, 16 patients were treated with non-platinum based chemotherapy. Finally, a total of 259 mUC patients were enrolled into our study.

### Assessment of TILs

**Figure 3:**
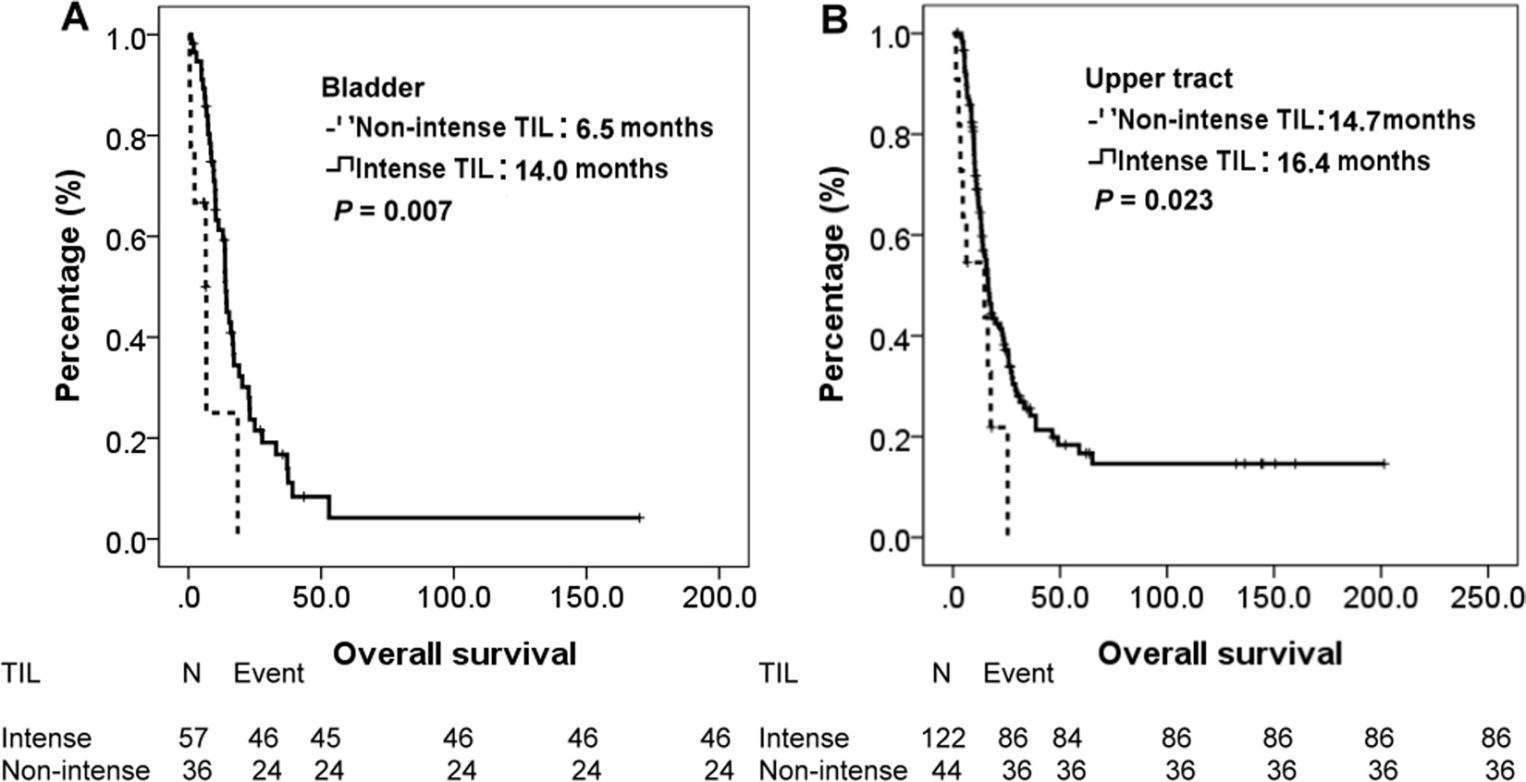
Representative urothelial carcinoma specimens stained with haematoxylin and eosin (H&E 100X) demonstrating examples with (**A**) intense intratumoral tumor infiltrating lymphocytes (itTILs) (black arrows), (**B**) intense stromal tumor infiltrating lymphocytes (sTILs) (black arrows).

Full-face hematoxilin and eosin stained (HES) slides of primary tumors were retrieved for the evaluation of percentage of TILs. The analysis was based on the recommendations by an International TILs Working Group 2014^40^. All pathologic slides were evaluated by two dedicated urologic pathologists (H.S. Huang and P.H. Lee) independently, who were blinded to clinical information, including treatment allocation and outcomes. Our patients were stratified according to the percentage of TILs labeled as intense TILs and non-intense TILs, respectively. Intense TILs was defined as more than 10% of lymphocyte infiltration, while non-intense TILs was defined as less than 10% infiltration. According to the recommendations by the international TILs Working Group 2014, no formal recommendation for a clinically relevant TILs threshold can be given at this stage. Therefore, we set the cut-off value at 10% according to previous literatures^41^. TILs was also classified into intratumoral (itTILs) and stromal (sTILs) according to their predominant area of infiltration. itTILs were defined as lymphocytes infiltrating within tumor nests and in direct contact with the tumor cells, whereas sTILs were defined as lymphocytes infiltration surrounding tumor stroma. Figure 3 exhibited the picture of intense itTILs and intense sTILs, respectively.

### Statistical analysis

Patients were divided according to the percentage of TILs. All the clinical characteristics were analyzed with Pearson χ^2^ test between both groups. Survival outcomes were presented with Kaplan-Meier curves to estimate overall survival (OS) of all patients. Subgroup analysis was also performed according to location of primary tumor, labeling as upper tract urothelial carcinoma (UTUC) and urothelial carcinoma of bladder (UCB). OS was calculated from the diagnosis of metastatic disease until the date of death or the last contact when the patients were still alive at the time of the follow-up visit. Univariable and multivariable Cox regression analysis were constructed for survival and presented with hazard ratio (HR) and 95% confidence intervals (CIs). All statistical tests were two-sided. *P*-values < 0.05 were considered to be statistically significant.

## Electronic supplementary material

Supplementary Figure 1.
