# Supplementary material for: Prognostic impact of tumor infiltrating lymphocytes on patients with metastatic urothelial carcinoma receiving platinum based chemotherapy
Source: Sci Rep. 2018 May 10;8:7485. doi: 10.1038/s41598-018-25944-1 (PMC5945658; doi:10.1038/s41598-018-25944-1)
Supplement: Supplementary file 1 — Supplementary Figure 1. [file 41598_2018_25944_MOESM1_ESM.pdf]

Supplementary figure of Original article

Title: Prognostic impact of tumor infiltrating lymphocytes on patients with metastatic urothelial carcinoma receiving platinum based chemotherapy

Author list: Hui-Shan Huang M.D.[1], Harvey Yu-Li Su M.D.[2], Pei-Hsu Li M.D.[1], Po-Hui Chiang M.D.,Ph.D.[3] ,Cheng-Hua Huang M.D.[2], Chien-Hsu Chen M.D.[3], Meng-Che Hsieh M.D.[2]

Affiliation:

1. Department of Pathology, Kaohsiung Chang Gung Memorial Hospital and Chang Gung University College of Medicine, Kaohsiung, Taiwan
2. Division of Hematology-Oncology, Department of Internal Medicine, Kaohsiung Chang Gung Memorial Hospital and Chang Gung University College of Medicine, Kaohsiung, Taiwan
3. Division of Urology, Department of Surgery, Kaohsiung Chang Gung Memorial Hospital and Chang Gung University College of Medicine, Kaohsiung, Taiwan

Address: No.123, DaPi Rd. NiaSong Dist, Kaohsiung City 83301, Taiwan

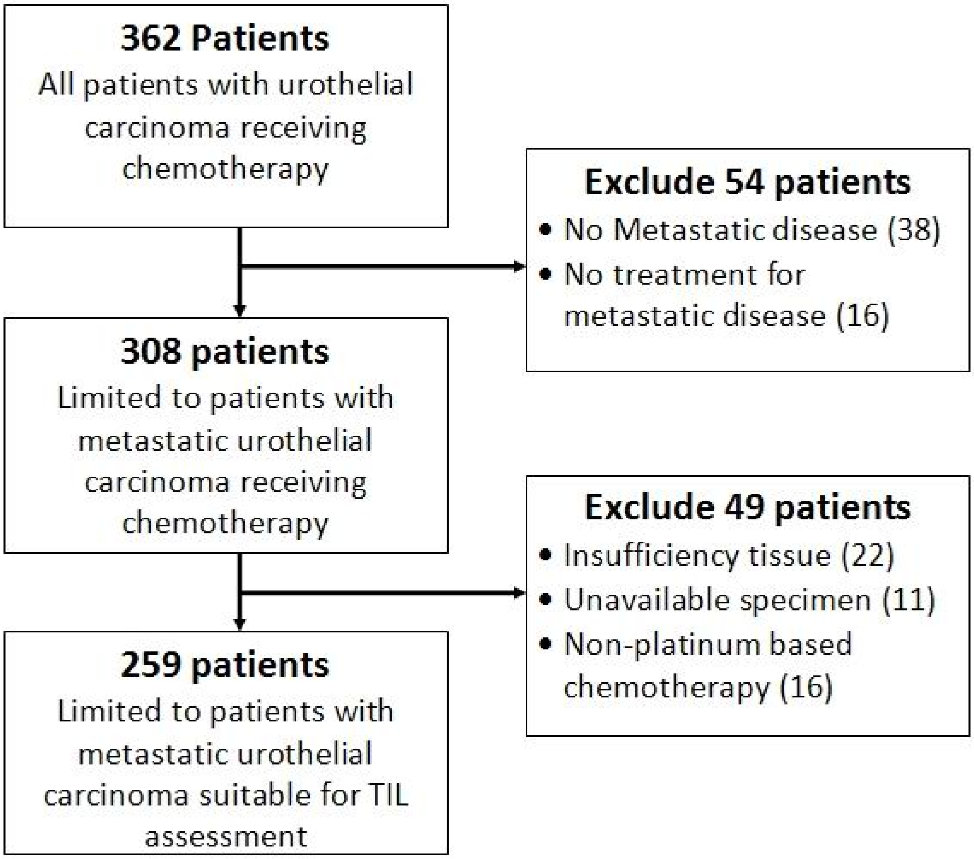

Supplementary Figure 1. The consort flow diagram of our study
